# Effective 10-MDP Bonding to Saliva-Contaminated Dentin

**DOI:** 10.3290/j.jad.c_1966

**Published:** 2025-04-16

**Authors:** Line Etiennot, Michiel Ordies, Chenmin Yao, Ben Mercelis, Marleen Peumans, Bart Van Meerbeek

**Affiliations:** a Line Etiennot PhD Student, KU Leuven (University of Leuven), Department of Oral Health Sciences, BIOMAT & UZ Leuven (University Hospitals Leuven), Dentistry. Kapucijnenvoer 7, Blok a – box 7001, BE-3000 Leuven, Belgium. Data curation, performed the statistical analysis and wrote the manuscript.; b Michiel Ordies Master in Specialized Oral Health, Restorative Dentistry, KU Leuven (University of Leuven), Department of Oral Health Sciences, BIOMAT & UZ Leuven (University Hospitals Leuven), Dentistry. Kapucijnenvoer 7, Blok a – box 7001, BE-3000 Leuven, Belgium. Data curation, experimental design, and performed the experiments.; c Chenmin Yao Postdoctoral Researcher, KU Leuven (University of Leuven), Department of Oral Health Sciences, BIOMAT & UZ Leuven (University Hospitals Leuven), Dentistry. Kapucijnenvoer 7, Blok a – box 7001, BE-3000 Leuven, Belgium. Idea, contributed to the experimental design, consulted in the methodology, data curation, and contributed to the discussion.; d; e Ben Mercelis Research Group Manager, KU Leuven (University of Leuven), Department of Oral Health Sciences, BIOMAT & UZ Leuven (University Hospitals Leuven), Dentistry. Kapucijnenvoer 7, Blok a – box 7001, BE-3000 Leuven, Belgium. Consulted on the methodology and performed part of the experiments.; f Marleen Peumans Professor, KU Leuven (University of Leuven), Department of Oral Health Sciences, BIOMAT & UZ Leuven (University Hospitals Leuven), Dentistry. Kapucijnenvoer 7, Blok a – box 7001, BE-3000 Leuven, Belgium. Supervised and contributed to experimental design.; g Bart Van Meerbeek Full Professor, KU Leuven (University of Leuven), Department of Oral Health Sciences, BIOMAT & UZ Leuven (University Hospitals Leuven), Dentistry. Kapucijnenvoer 7, Blok a – box 7001, BE-3000 Leuven, Belgium. Idea, supervision, proofread the manuscript, and contributed to the discussion.

**Keywords:** adhesive, bond strength, decontamination, Katana Cleaner, saliva

## Abstract

**Purpose:**

The study aimed to measure the efficacy of 10-methacryloyloxydecyl dihydrogen phosphate (10-MDP) saliva-decontamination protocols by measuring bonding effectiveness to saliva-contaminated dentin following different surface-decontamination protocols.

**Materials and Methods:**

The micro-tensile bond strength (µTBS) of the two-step self-etch (SE) adhesive Clearfil SE Bond 2 (‘CSE2’, Kuraray Noritake) and the one-step SE adhesive Clearfil Universal Bond Quick (‘CUBQ’, Kuraray Noritake) to saliva-contaminated bur-cut dentin was measured when saliva-contaminated dentin was decontaminated by either the 10-MDP-containing Katana Cleaner (‘KC’, Kuraray Noritake) or CSE2 primer (‘CSE2p’), with bonding to saliva-contaminated (‘saliva(–)’) and non-contaminated dentin (‘clean(+)’) having served as negative and positive control, respectively. Half of the specimens were subjected to µTBS testing ‘immediately’ after 1-week water storage, while the other ‘aged’ half was tested after 50,000 thermocycles. Statistics involved linear mixed modeling (LMM) with restricted maximum likelihood (REML) estimation (α = 0.05).

**Results:**

Overall, the two-step SE adhesive CSE2 outperformed the one-step SE adhesive CUBQ. Saliva-contaminated dentin was most effectively decontaminated when CSE2p was applied with both adhesives, closely followed by KC decontamination. Notably, CSE2 demonstrated satisfactory performance even without separate decontamination.

**Conclusion:**

Unaltered bonding to saliva-contaminated dentin was achieved upon surface decontamination with CSE2p and KC. Using CUBQ, additional decontamination with either CSE2p or KC is strongly recommended. In the case of CSE2, no additional decontamination agent is required.

Tooth tissue lost by decay, trauma, or erosion is preferably restored with resin-based composite (RBC) because of its tooth-colored appearance and bondability. This ensures excellent esthetic outcomes while allowing minimally invasive tooth restoration.^29,36^ Nevertheless, adhesion to tooth structure remains a highly sensitive process prone to errors. In that matter, contamination of the prepared tooth surface can compromise the bond strength of the adhesive and cause marginal leakage and discoloration, secondary caries, and eventually bulk failure of the restoration.^5,8,24^

Saliva, composed of over 99% water, is a common contaminant that reduces bond strength by acting as both a physical and chemical barrier.^21,27,33^ It lowers the dentin’s surface energy, preventing proper adhesive wetting and limiting the interaction with the dentin substrate.^5,8,33,34^ Additionally, saliva contains hydrolytic enzymes and immune mediators that can cause structural alterations in dentin.^5,12,21,33^ Therefore, it is essential in the clinical setting to avoid surface contamination to achieve optimal bond effectiveness.

Qualitative operative field isolation can clinically best be obtained through the placement of a rubber dam. When properly positioned, it ensures complete field isolation and allows adhesive procedures to be performed with minimal risk of surface contamination.^28,30,35^ However, achieving perfect, leak-free isolation can sometimes be challenging in clinical situations. In the event of contamination, a rigorous decontamination protocol should be applied to restore the bond’s quality. Currently, it is recommended to accomplish this by rinsing with water, which is a critical step, followed by drying and repeating the adhesive procedure.^5,8,12,32^ However, this method depends on various factors, such as the type of adhesive used. As a result, there is no conclusive evidence that the bond strength will fully have been restored after this decontamination protocol, which clinically is also time-consuming. ^8,23^

To address this issue, decontamination agents have been marketed, such as Ivoclean (Ivoclar; Schaan, Liechtenstein) and Katana Cleaner (‘KC’; Kuraray Noritake; Tokyo, Japan). Ivoclean (Ivoclar) is primarily indicated for extraoral cleaning of pre-treated ceramic and metal restoration surfaces that have been contaminated during intraoral try-in.^13^ KC offers a wider range of surface-decontamination applications, including extraoral uses such as decontamination of posts, ceramics, and resin-based semi-direct/indirect restorations, as well as intraoral applications like cleaning cavities, root canals, and abutments. Current literature supports the effectiveness of KC in decontaminating dentin, zirconia, and other surfaces.^4,14,16,31,33^ According to the manufacturer, this versatility is attributed to the presence of the functional monomer 10-methacryloyloxydecyl dihydrogen phosphate (10-MDP).^17^

Today, 10-MDP is well-known for its beneficial role in dental adhesives and is considered one of the most effective functional monomers to bond to the challenging dentin substrate.^7,10,11,22^ Most of the recent generations of ‘universal adhesives’ contain 10-MDP.^22^ It is known to establish a strong and stable chemical bond with hydroxyapatite by forming water-insoluble 10-MDP_Ca salts, a process known as nano-layering, which results in a durable bond resistant to (bio)degradation.^7,10^ Beyond its adhesive properties, 10-MDP’s unique chemical structure, featuring hydrophilic and hydrophobic groups at opposite ends (Fig 1), allows it to interact with saliva and other contaminants, effectively disrupting their adhesion to the tooth surface.^22,31^ The combination of 10-MDP and water creates a strong surfactant effect in decontamination agents such as KC. When applied to the contaminated tooth surface, the hydrophobic group of 10-MDP binds to the organic components of saliva, forming micelles that encapsulate the contaminant. This mechanism reduces the surface tension of the contaminants, disrupts their adhesion, and facilitates their removal with rinsing. Importantly, any residual 10-MDP remaining at the bonding interface after rinsing is not a concern. Instead, it contributes positively to the bonding process.^14,31,33^ Thus, KC’s innovative formulation takes advantage of the multifunctional properties of 10-MDP to not only decontaminate dental surfaces effectively but also improve adhesive performance, ensuring durable and reliable outcomes.

**Fig 1:**
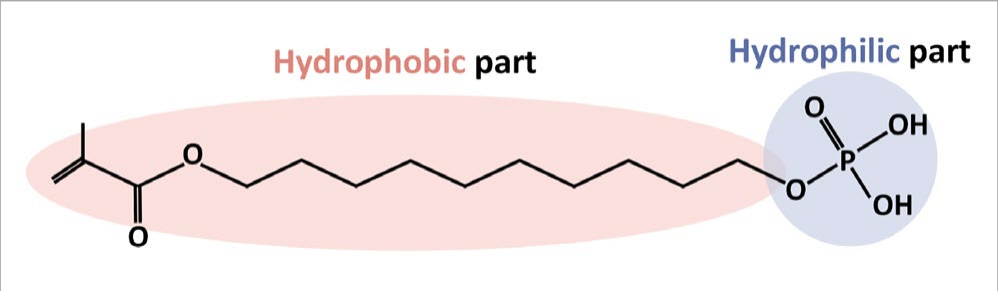
Chemical structure of the functional monomer 10-methacryloyloxydecyl dihydrogen phosphate (10-MDP) featuring distinct hydrophilic (phosphate group) and hydrophobic (methacrylate group) regions separated by a long carbon chain (spacer). This arrangement is key to its unique adhesive properties in dental applications.

Similarly, the cleaning efficacy of experimental decontamination cleaners containing 10-MDP has been investigated in the literature, demonstrating satisfactory results.^16,31^ Likewise, CSE2p (Kuraray Noritake), which is part of the two-step self-etch (2-SE) adhesive Clearfil SE Bond 2 (‘CSE2’; Kuraray Noritake) and is regarded as gold standard in dental adhesion, also contains 10-MDP and water, making it possibly a suitable material for surface decontamination.^22^

The aim of this study was, therefore, to evaluate the saliva- decontamination efficiency of the novel 10-MDP-based decontamination agent, ‘KC’, in comparison to that of the 10-MDP-based CSE2p. The micro-tensile bond strength (µTBS) was evaluated of saliva-contaminated versus non-contaminated dentin bonded with the market-representative 2-SE adhesive CSE2p and its simplified one-step universal adhesive (1-UA) Clearfil Universal Bond Quick (‘CUBQ’; Kuraray Noritake). Half of the specimens were subjected to µTBS testing ‘immediately’ after 1-week storage in water at 37°C. The other ‘aged’ half was tested after 50,000 thermocycles. The null hypothesis tested was that KC and CSE2p restored the µTBS of both adhesives following saliva contamination before and after aging.

## MATERIALS AND METHODS

### Dentin-specimen Preparation

**Fig 2:**
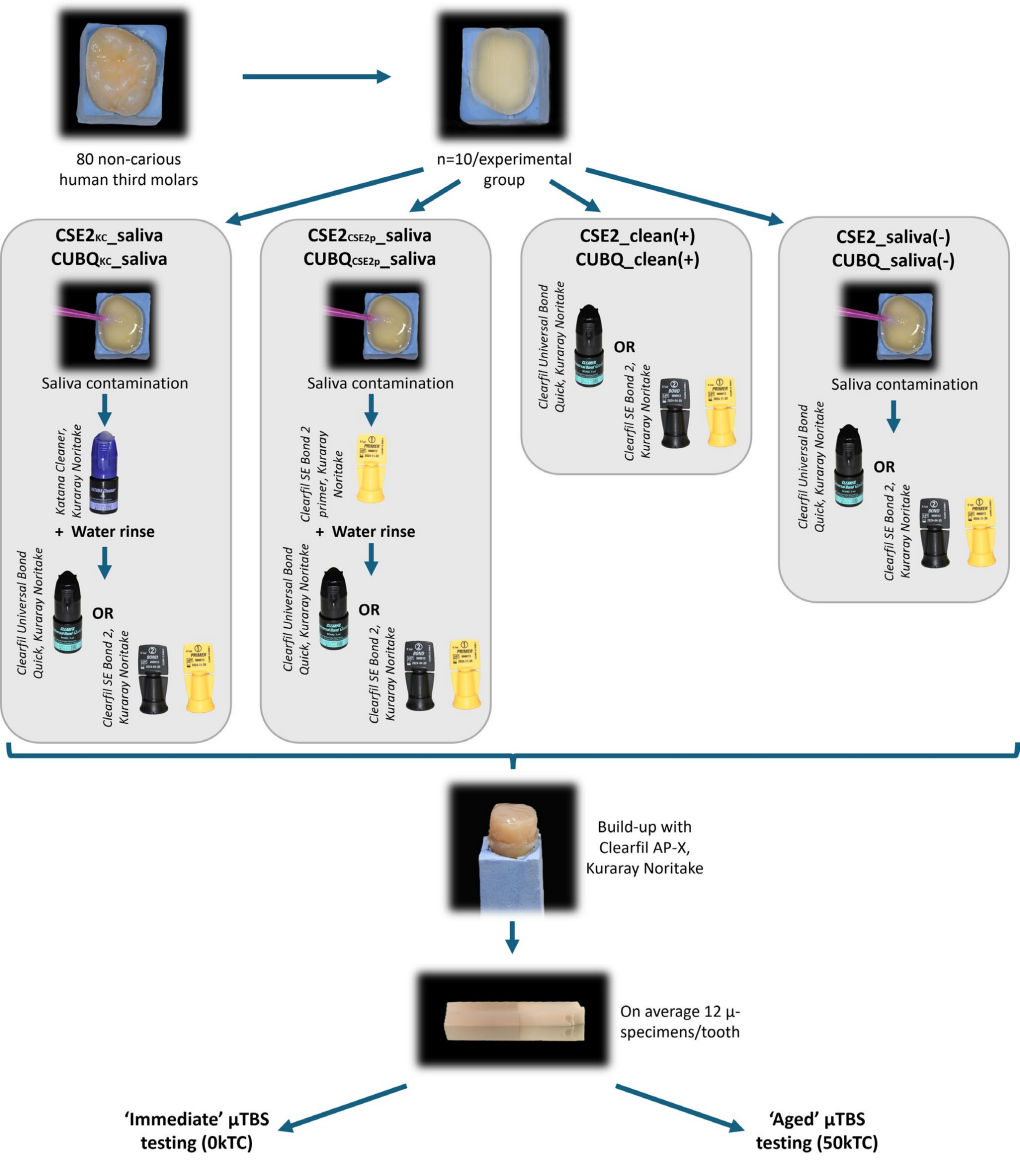
Graphical presentation of the experimental procedure. Eighty non-carious human third molars were randomly subdivided into eight experimental groups (n = 10/experimental group). The eight groups were categorized as either ‘CSE2_KC__saliva’ and ‘CUBQ_KC__saliva’ when the two adhesives were applied on saliva-contaminated dentin upon saliva decontamination using Katana Cleaner (‘KC’; Kuraray Noritake), as ‘CSE2_CSE2p__saliva’ and ‘CUBQ_CSE2p__saliva’ when the two adhesives were applied on saliva-contaminated dentin upon saliva decontamination using Clearfil SE Bond 2 primer (‘CSE2p’; Kuraray Noritake), as ‘CSE2_clean(+)’ and ‘CUBQ_clean(+)’ when the adhesives were applied to non-contaminated dentin (positive control), or as ‘CSE2_saliva(–)’ and ‘CUBQ_saliva(–)’ when the adhesives were applied to saliva-contaminated dentin without decontamination (negative control).

A graphical presentation of the experimental procedure is shown in Figure 2. Informed consent was obtained from the Commission of Medical Ethics to collect 80 non-carious human third molars, randomly subdivided into eight experimental groups (n = 10/experimental group). The teeth were preserved in a 0.5% chloramine T solution mixed with water and were utilized within six months following their extraction. The occlusal third of the crown was removed at the level of mid-coronal dentin using a slow-speed diamond saw (Micracut 151, Metkon; Bursa, Turkey), while the roots were fixed in a small gypsum block to ease manipulation. To create a standard smear layer on dentin, a MicroSpecimen Former (University of Iowa; Iowa, IA, USA) equipped with a high-speed medium-grit (107 µm) diamond bur (882, Komet; Lemgo, Germany) was used. After preparation, the exposed dentin surfaces were examined by stereo-microscopy (Stemi 2000-CS, Zeiss; Oberkochen, Germany) to check for enamel remnants or potential pulp exposures. In the case of enamel remnants, the dentin surface was further ground by bur; in the case of pulp exposure, the specimen was discarded.

Before applying the adhesive protocol, all specimens were placed for 30 min in an incubator at 37°C with 100% humidity. To contaminate dentin, ‘fresh’ human saliva collected from one individual was applied using a micro-brush with a gentle brushing motion for 15 s. The saliva film was then gently air-thinned for 10 s until it became a glossy, motionless layer, after which it was left untouched for 60 s. Depending on the group, KC and CSE2p were applied following the manufacturer’s instructions for the KC agent. This involved applying the product with a rubbing motion for 10 s, then rinsing and air-drying for 5 s. Similarly, the two adhesives, CSE2 and CUBQ, were applied according to the respective manufacturer’s instructions outlined in Table 1. After the adhesive treatment, a 5-mm build-up was made using the micro-hybrid composite Clearfil AP-X (Kuraray Noritake) in increments of a maximum of 2 mm, with each increment light-cured for 20 s. Light-curing was performed using the LED light-curing unit (LCU) SmartLite Pro (Dentsply Sirona; Konstanz, Germany) with a light output of ≥ 1250 mW/cm^2^. The light output was checked before and after specimen preparation using a Marc Resin Calibrator (BlueLight Analytics; Halifax, Canada).

**Table 1:** Composition and application instructions of the materials used in this study

Material	Composition^1^	Instructions
**Clearfil SE Bond 2** [CS2] (Kuraray Noritake)	**Primer:** 10-MDP, HEMA, hydrophilic aliphatic dimethacrylate, camphorquinone, water **Adhesive:** 10-MDP, HEMA, BisGMA, hydrophobic aliphatic dimethacrylate, camphorquinone, initiators, accelerators, silanated colloidal silica	1. Apply CSE2 primer using a micro-brush and leave it in place for 20 s before mild air-drying for more than 5 s. 2. Apply CSE2 adhesive, followed by gentle air-drying for 3 s. 3. Light-cure using a high-power LED light-curing unit for 10 s.
**Clearfil Universal Bond Quick** [CUBQ] (Kuraray Noritake)	BisGMA, ethanol, HEMA, 10-MDP, hydrophilic amide monomer, colloidal silica, silane coupling agent, sodium fluoride, camphorquinone, water	1. Apply in a rubbing motion, no waiting. 2. Mildly air-dry (≥5 s) until the adhesive no longer moves. 3. Light-cure using a high-power LED light-curing unit for 10 s.
**Katana Cleaner** [KC] (Kuraray Noritake)	10-MDP, water, PEG, accelerator, dyes, TEA	1. Apply the cleaning agent with a rubbing motion for 10 s. 2. Rinse with water and air-dry for 5 s.
**Clearfil AP-X** (Kuraray Noritake)	Filler: Silanated barium glass filler, silanated silica filler, silanated colloidal silica Monomers: BisGMA, TEGDMA, camphorquinone	1. Apply the restorative composite in layers of a maximum of 2 mm until a height of 5 mm is reached. 2. Light-cure each 2-mm layer for 20 s with a high-power LED light-curing unit.
^1^10-MDP: 10-methacryloyloxydecyl dihydrogen phosphate; HEMA: 2-hydroxyethyl methacrylate; BisGMA: bisphenol A glycidyl methacrylate; PEG: polyethyleneglycol; TEA: triethanolamine; TEGDMA: triethylene glycol dimethacrylate.

Subsequently, the build-up specimens were kept for 24 h in an incubator at 37°C and 100% humidity before being immersed in distilled water at 37°C for 6 days. After 1 week, all specimens were sectioned perpendicular to the interface using a water-cooled diamond saw (Accutom-50, Struers; Ballerup, Denmark) to achieve, on average, 12 rectangular sticks with 1×1×8 mm (µ-specimen) dimensions. The eight groups were categorized as follows: both adhesives were applied upon saliva decontamination using KC (‘CSE2_KC__saliva’; ‘CUBQ_KC__saliva’) and upon the additional use of CSE2p (‘CSE2_CSE2p__saliva’; ‘CUBQ_CSE2p__saliva’). Additionally, each adhesive involved a positive control (‘CSE2_clean(+)’; ‘CUBQ_clean(+)’) and a negative control (‘CSE2_saliva(–)’; ‘CUBQ_saliva(–)’).

### Micro-Tensile Bond Strength (µTBS) Testing

For each group (n = 10), about 60 µ-specimens (6 µ-specimens per tooth, split-tooth design) were tested ‘immediately’ to determine the ‘immediate’ (‘0kTC’) µTBS. The other 60 µ-specimens were subjected to 50,000 thermocycles between two water baths at 5°C and 55°C using a THE-1200 thermocycler (SD Mechatronik; Munich, Germany) before testing to measure the ‘aged’ (‘50kTC’) µTBS.

The µTBS test was performed using an LRX testing device (LRX, Lloyd; Hampshire, UK). The specimens were initially attached to a BIOMAT jig with a cyanoacrylate-based two-component glue (Model Repair II Blue, Dentsply Sirona Sankin; Tochigiken, Japan) and subjected to stress at a crosshead speed of 1 mm/min until fracture and using a load cell of 100 N. Specimens that failed before the actual test were recorded as pre-test failures (ptf) and assigned a value of 0 MPa for the calculation of the mean µTBS. Specimens that fractured due to handling errors were recorded as manipulation errors and excluded. The µTBS of all µ-specimens originating from each tooth were averaged, with statistical analysis conducted on the 10 tooth-based µTBS averages rather than on all individual specimen values. Statistical analysis involved a linear mixed model (LMM) with REML estimation at a significance level of α = 0.05 to account for fixed effects of ‘adhesive’, ‘application protocol’, and ‘aging’, as well as random effects due to variability among teeth.

After fracture, the specimens were evaluated using a stereomicroscope (Stemi 2000-CS, Zeiss; Oberkochen, Germany) at 50× magnification to classify the failure mode as either ‘cohesive failure in dentin’, ‘cohesive failure in composite’, ‘adhesive (interfacial) failure’, or ‘mixed failure including the adhesive interface’.

### SEM Failure-Mode Analysis

Representative fractured surfaces, showing the most common failure mode and originating from specimens with a µTBS recorded near the mean or from ptf specimens, were selected for scanning electron microscopy (SEM) (JSM-6610LV, Jeol; Tokyo, Japan). After fixation using 2.5% glutaraldehyde, the specimens were gradually dehydrated in ethanol and dried with hexamethyldisilazane (Acros Organics, Thermos Fisher Scientific; Geel, Belgium). Next, specimens were gold-sputtered (JFC-1300, Jeol) before being examined using SEM.

## RESULTS

### Bonding Effectiveness to Flat Dentin After Different Saliva Surface-Decontamination Protocols

**Fig 3:**
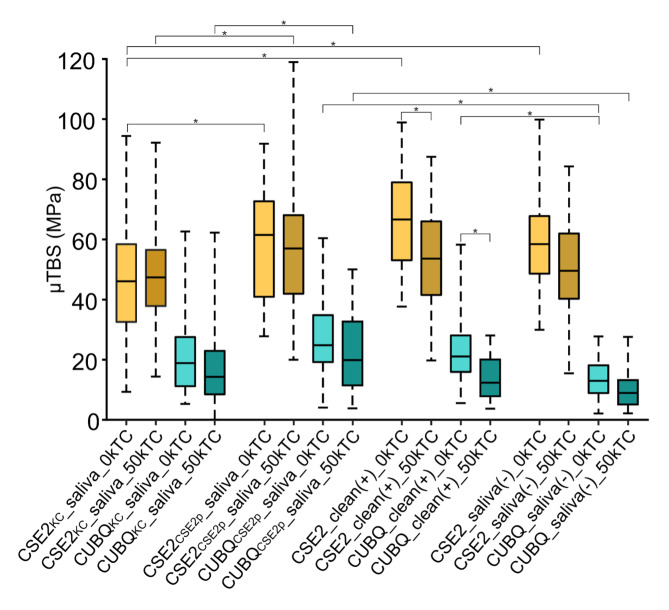
Box-and-whisker plots of the ‘immediate’ 0 kTC and ‘aged’ 50 kTC-aged µTBS (in MPa) of the CSE2 and CUBQ adhesives when applied to saliva-contaminated dentin following the different decontamination protocols investigated. Saliva-contaminated dentin was decontaminated by Katana Cleaner (KC) or CSE2 primer (CSE2p). The negative control involved bonding without decontamination (saliva(–)) versus bonding to non-contaminated dentin (clean(+)) as the positive control. The thick horizontal line within each box represents the median µTBS. The horizontal lines in each box represent, from top to bottom, the maximum µTBS, the upper quartile, the median µTBS, the lower quartile, and the minimum µTBS measured for each experimental group. Statistically significant differences in µTBS are connected by horizontal lines and indicated with an asterisk, except for the differences between the CSE2 and CUBQ µTBSs, which are all significantly different.

All µTBS data are detailed in Table 2 and graphically represented in Figure 3. Overall, a significant difference was observed between the two adhesives, irrespective of their application protocol, both before and after aging, with CSE2 as a 2-SE adhesive having revealed significantly higher µTBS than the 1-UA CUBQ following all experimental conditions. Additionally, no difference in µTBS was observed before (0k TC) and after aging (50 kTC) for the two adhesives, except for the positive controls CSE2_clean(+) and CUBQ_clean(+) that revealed a relatively slight decrease in µTBS upon aging. In the CSE2 adhesive group at 0 kTC, the µTBS for CSE2_KC__saliva was significantly lower compared to the other application protocols. At 50 kTC, CSE2_KC__saliva remained significantly lower when compared to CSE2_CSE2p__saliva. In the CUBQ adhesive group at 0 kTC, CUBQ_saliva(–) had a significantly lower µTBS than CUBQ_CSE2p__saliva and CUBQ_clean(+). At 50 kTC, the highest µTBS was recorded for CUBQ_CSE2p__saliva, significantly outperforming CUBQ_KC__saliva and CUBQ_saliva(–). Only three ptfs were recorded for CUBQ_KC__saliva.

**Table 2:** Micro-tensile bond strength (µTBS) of the adhesives CSE2 and CUBQ to flat dentin

Clearfil SE Bond 2 (CSE2)
µTBS (MPa)*	CSE2_KC__saliva	CSE2_CSE2p__saliva	CSE2_clean(+)	CSE2_saliva(–)
Clearfil Universal Bond Quick (CUBQ)
µTBS (MPa)*	CUBQ_KC__saliva	CUBQ_CSE2p__saliva	CUBQ_clean(+)	CUBQ_saliva(–)
0 kTC	44.1 ± 19.8 (0/50)	58.5 ± 18.3 (0/31)	66.5 ± 15 (0/56)	58.7 ± 14.9 (0/57)
50 kTC	47.9 ± 15.3 (0/59)	57.4 ± 20.3 (0/43)	54.9 ± 16.5 (0/59)	50.6 ± 16.8 (0/55)
0 kTC	21.3 ± 12.7 (0/58)	27.2 ± 11.5 (0/60)	22.8 ± 10.5 (0/58)	13.1 ± 6.6 (0/60)
50 kTC	16.8 ± 11.2 (3/56)	22.2 ± 10.8 (0/59)	13.8 ± 6.6 (0/60)	9.5 ± 5.7 (0/60)
Mean ± SD (ptf/n); SD: standard deviation; ptf: pre-test failure; n: total number of µ-specimens including ptfs. KC: Katana Cleaner; CSE2p: Clearfil SE Bond 2 primer.

As indicated in Table 3, the statistical results of the LMM revealed significant effects for the three variables ‘Adhesive’, ‘Application protocol’, and ‘Aging’. Additionally, all interactions were significant except for the ‘Adhesive × Aging’ interaction, suggesting that aging does not affect the adhesive’s impact. In contrast, the results indicate that the effect of the application protocol is dependent on the selected adhesive and is also influenced by the aging conditions.

**Table 3:** Statistical analysis of the fixed variables and interactions of LMM

	numDF	denDF	F-value	P-value
Adhesive	1	460	1396	<0.0001*
Application protocol	3	460	21.55	<0.0001*
Aging	1	408	33.27	<0.0001*
Adhesive × Application protocol	3	460	15.68	<0.0001*
Application protocol × Aging	3	408	5.77	0.0007*
Adhesive × Aging	1	408	0.68	0.4103
Application protocol × Adhesive × Aging	3	408	3.376	0.0184*
*Statistically significant.

**Fig 4:**
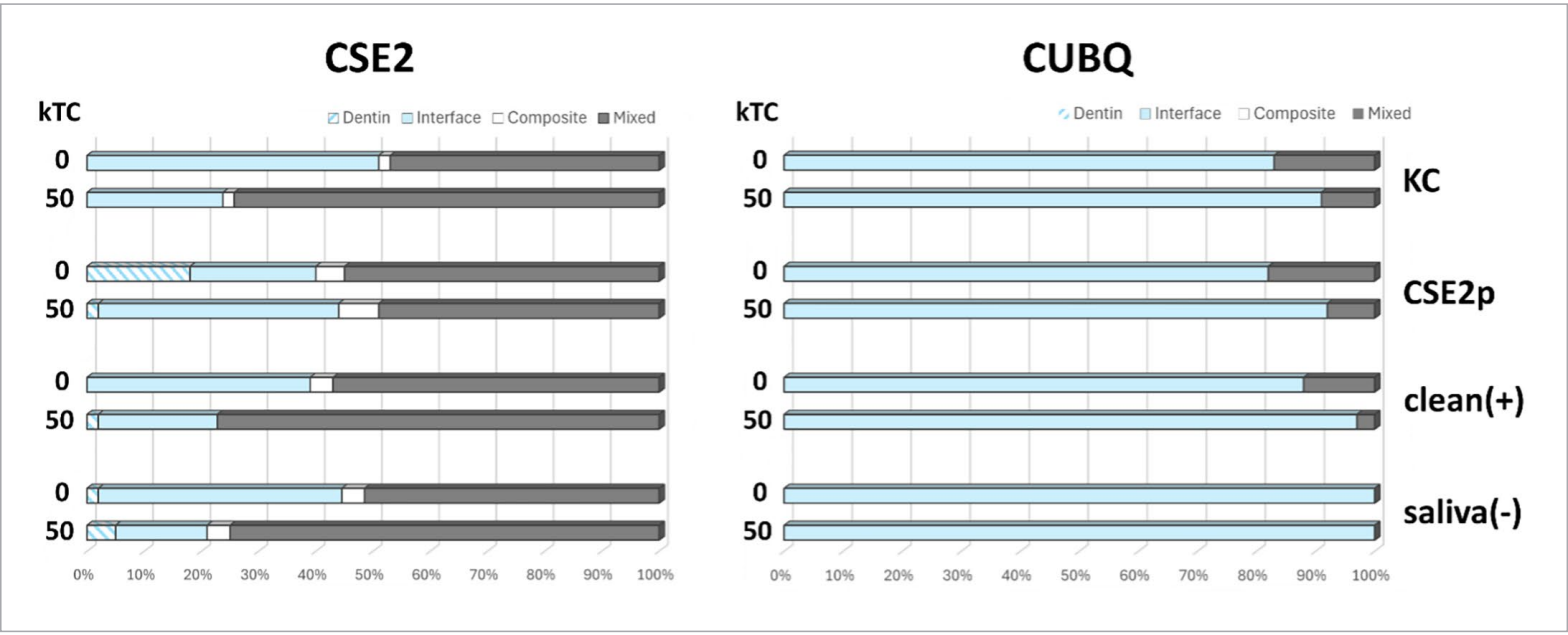
Failure-mode distribution of the CSE2 and CUBQ µ-specimens upon µTBS testing after bonding to saliva-contaminated dentin following different application protocols. KC: Katana Cleaner; CSE2p: CSE2 primer; clean(+): positive control; saliva(–): negative control. Dentin: cohesive failure in dentin; Interface: adhesive interfacial failure; Composite: cohesive failure in composite; Mixed: mixed failure.

The failure-mode distribution is presented in Figure 4. The failure patterns were quite similar across both adhesives. In the CSE2 adhesive group, the majority were mixed failures, including the adhesive interface, followed by failures at the adhesive-dentin interface. In the CUBQ adhesive group, most failures occurred at the adhesive-dentin interface.

**Fig 5:**
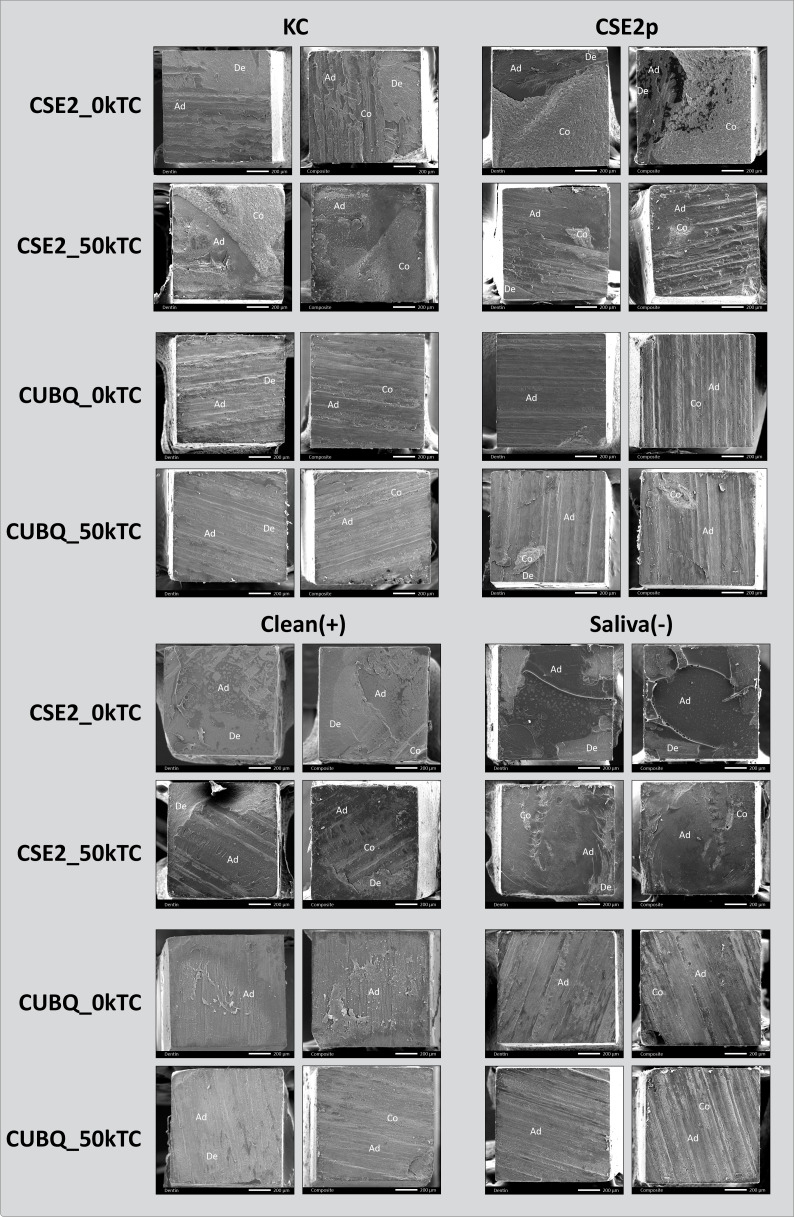
Representative SEM photomicrographs of µ-specimens (dentin and composite side), illustrating the failure modes for CSE2 and CUBQ after bonding to saliva-contaminated dentin following the different decontamination protocols investigated. The CSE2 groups at 0 kTC and 50 kTC revealed primarily ‘adhesive (interfacial) failure’ and ‘mixed failure including the adhesive interface’ failure modes. The CUBQ groups at 0 ktC and 50 kTC presented a majority of ‘adhesive (interfacial) failures’. KC: Katana Cleaner; CSE2p: CSE2 primer; clean(+): positive control; saliva(–): negative control; De: dentin; Ad: adhesive resin; Co: composite.

Representative SEM photomicrographs of fractured µ-specimens are presented in Figure 5. The CSE2 adhesive group primarily exhibits a combination of mixed and interface failure modes. In contrast, the CUBQ adhesive group predominantly shows interface failure modes.

## DISCUSSION

According to the literature, salivary contamination can compromise adhesion to both enamel and dentin, with more studies indicating an adverse effect on dentin.^8,21^ Additionally, SE adhesives are reported to maintain µTBS better than etch-and-rinse (E&R) adhesives in the presence of saliva.^8^ It has been suggested that the acidic primer of SE adhesives can degrade saliva proteins, resulting in a beneficial effect on bonding. In contrast, for E&R adhesives, phosphoric acid can over-etch dentin and expose collagen fibers, making the structure susceptible to enzymatic degradation by saliva, which negatively impacts bonding.^5,8^ Therefore, it is even recommended to avoid E&R adhesives when bonding to saliva-contaminated dentin.^8^

The present study evaluated the effectiveness of one decontamination agent (KC) and the additional use of a 10-MDP primer (CSE2p) on the bonding effectiveness of a market-representative and considered gold standard 2-SE adhesive (CSE2) versus a market-representative 1-UA adhesive (CUBQ), both containing 10-MDP, to saliva-contaminated dentin, as assessed in terms of µTBS.

CSE2 demonstrated higher µTBS compared to the 1-UA CUBQ applied in SE mode. These findings are consistent with the literature and are also reflected in the higher prevalence of adhesive interface failure modes observed in the CUBQ group.^2,15^ Both CSE2 and CUBQ adhesives, manufactured by Kuraray Noritake, contain 10-MDP as a functional monomer, which was first introduced by the company in 1983.^18^ As a result, the 10-MDP in both adhesives is expected to be of consistent quality, a critical factor for optimal adhesive performance.^22^ Accordingly, the findings of this study support existing evidence that the separate application of a 10-MDP-containing primer followed by a hydrophobic adhesive resin with a relatively thick film thickness positively impacts adhesion.^2^ Indeed, incorporating a separate hydrophobic layer can enhance bonding performance. This improvement has been observed across diverse adhesives but appears particularly advantageous for UAs due to their very low film thickness. This characteristic makes these adhesives more susceptible to oxygen inhibition during polymerization, rendering them more prone to water-related degradation.^1,6,9,19,22^ In the experimental setup of our study, no separate (additional) hydrophobic layer was used for both adhesives. However, it is plausible to speculate that the CUBQ group might have achieved better results if an additional layer, such as a flowable composite, had been applied over the adhesive.

Regarding aging of the samples, all groups exhibited a trend of reduced µTBS after aging (50 kTC), which aligns with expectations, considering that the 50,000 thermocycles represented a significantly greater level of aging compared to the 500 thermocycles specified in the ISO/TS 4660 standard,^25^ and even the 10,000 thermocycles recommended in the Academy of Dental Materials guidelines.^3^ Additionally, µ-specimens were aged, involving 50-day direct exposure of the adhesive interface to water, and not bulk specimens as for shear bond-strength testing as described in the ISO Standard 29022 for dental adhesives.^26^ Nevertheless, only for the positive controls, CSE2_clean(+) and CUBQ_clean(+), was the rather slight reduction in µTBS between 0 kTC and 50 kTC significant. However, this significance level should be interpreted with some caution, as these values align closely with the overall bonding effectiveness results recorded for both adhesives and CSE2 in particular.

Using CUBQ, the negative control (CUBQ_saliva(–)) revealed the lowest µTBS recorded, strengthening the knowledge that saliva contamination negatively affects bonding to tooth structure and dentin in particular. Overall, CUBQ_KC__saliva performed adequately, as the results were consistent with the positive control (CUBQ_clean(+)), both before and after aging. However, CUBQ_CSE2p__saliva still outperformed the other CUBQ groups. This suggests that applying the CSE2p not only decontaminated the dentin surface effectively but also improved the bonding capability of CUBQ. In addition to compositional differences between KC and CSE2p, a plausible explanation could be that the CSE2p, besides surface decontamination, also partially demineralized (self-etched) and primed (adhesion-promoted) the dentin surface. This process likely facilitated enhanced penetration of CUBQ, leading to a stronger bond. However, combining the CSE2p with the CUBQ application introduced an extra step, leading back to the 2-SE adhesive concept and nullifying the time-saving advantage of 1-UAs. Additionally, adding the CSE2p to the CUBQ adhesive protocol did not yield results similar to those achieved by the CSE2_clean(+) control group, emphasizing that the 1-UAs cannot perform as well as a multi-step adhesive with a separate application of a primer and adhesive resin.

Using CSE2, CSE2_KC__saliva exhibited the lowest immediate and aged µTBSs, even when compared to the negative control CSE2_saliva(–). After aging, the CSE2_KC__saliva µTBS was only significantly lower compared to that recorded for CSE2_CSE2p__saliva. These results suggest that CSE2p could be an effective decontamination agent and, in our research, even slightly outperformed KC. Interestingly, the results also highlight that the CSE2 adhesive itself handled saliva contamination effectively, this when applied following the manufacturer’s instructions and without any additional surface-decontamination action. One possible explanation for these results could be the presence of 10-MDP in the aqueous CSE2p solution, similar to KC’s composition. The mild but lower acidity of CSE2 (pH = 2), as compared to that of KC (pH = 4.5), may even better degrade salivary proteins.^8,20^ In this sense, the decontamination properties of KC are likely primarily attributed to the 10-MDP component.^17^

Additionally, following contamination, a recent systematic review by Bourgi et al (2023) advised rinsing with water, drying, and re-bonding. However, this process may not ensure the complete elimination of saliva contamination.^5^ In our study, a rinsing step after contamination was not included, while it could likely have been beneficial. Nonetheless, from a clinical standpoint, this would extend treatment time, which is ideally kept as short as possible. Therefore, a decontamination agent that eliminates the need for an additional rinsing step would be advantageous. As mentioned above, CSE2 even effectively dealt with saliva contamination without any additional action required.

From a clinical perspective, if saliva contamination occurs before the application of a UA, these results suggest that an additional cleaning step is strongly recommended. Overall, with only few specific statistical differences recorded, the null hypothesis that KC and CSE2p restored the µTBS of both adhesives following saliva contamination before and after aging could be accepted. Regarding the decontamination agent, although the CSE2p demonstrated higher µTBS values, KC could also be recommended due to its satisfactory outcome and more affordable market price. If saliva contamination occurs before the application of CSE2, this study revealed that an additional cleaning agent will not necessarily provide added value.

Like any laboratory research, also this study presents several limitations. First, as suggested in the literature, no additional hydrophobic layer was applied to the CUBQ group to improve bonding performance. While this may have increased the µTBS, it remains uncertain whether this procedure would have had a significant impact following saliva decontamination. Secondly, this study specifically examined the effect of saliva contamination. However, other contaminants, such as blood, hemostatic agents, gloves, and temporary cements, have been reported in the literature as factors that influence adhesion to the tooth structure. Further research is required to explore effective decontamination protocols for these scenarios and to determine whether KC and CSE2p demonstrate similar results under such conditions as those observed in this study.^5,8^ Similarly, this study focused on the decontamination of dentin. However, according to the manufacturer, KC is also designed to clean other materials like ceramics, zirconia, and metals.^17^ Further investigation is required to determine whether CSE2p exhibits the same decontamination efficiency on these materials as KC. Finally, the literature indicates that saliva contamination affects the bonding process differently at various stages. Contamination occurring after the application of adhesive or even after light-curing may lead to different outcomes compared to contamination before the adhesive procedure.^23,33^ Unfortunately, there is limited literature on this topic. Therefore, further research is needed to explore the impact of the moment of saliva contamination during the adhesive process.

## CONCLUSION

KC and CSE2p demonstrated satisfactory bonding effectiveness results as decontamination agents on saliva-contaminated dentin. The two-step self-etch adhesive Clearfil SE Bond 2 (Kuraray Noritake) showed the highest µTBS, regardless of its application protocol, compared to the one-step universal adhesive Clearfil Universal Bond Quick (Kuraray Noritake). The results suggest that when contamination occurs during CUBQ application, an additional decontamination step with either CSE2p or KC is strongly recommended. In contrast, for CSE2, the use of a decontamination agent did not result in significantly better outcomes compared to the control groups, highlighting the superior performance of CSE2 as adhesive.

### Clinical Relevance

Decontamination is recommended for UA applications on saliva-contaminated dentin. However, the two-step self-etch adhesive CSE2 demonstrated excellent performance without requiring additional treatment.

### Acknowledgments

We gratefully thank Kuraray Noritake for providing the materials employed and investigated.

### Informed Consent

Informed consent was obtained from the Commission of Medical Ethics at UZ/KU Leuven under file number S64350 to collect 80 non-carious human third molars.
